# Pelvic dose accumulation accuracy in a CBCT based online adaptive radiotherapy system

**DOI:** 10.1002/acm2.70160

**Published:** 2025-07-14

**Authors:** Mikel Byrne, Xenia Ray, Kelly Kisling, Ben Archibald‐Heeren, Robert Finnegan, Suhuai Luo, Trent Aland, Peter Greer

**Affiliations:** ^1^ Icon Group South Brisbane QLD Australia; ^2^ School of Information and Physical Sciences University of Newcastle Newcastle NSW Australia; ^3^ Sydney Adventist Hospital Wahroonga NSW Australia; ^4^ Department of Radiation Medicine and Applied Sciences UC San Diego Health San Diego California USA; ^5^ Northern Sydney Cancer Centre Royal North Shore Hospital St Leonards NSW Australia; ^6^ Calvary Mater Newcastle Hospital Newcastle NSW Australia

**Keywords:** deformation, delivered dose, dose accumulation, ethos, OART, online adaptive, radiotherapy

## Abstract

**Introduction:**

Accurate dose accumulation is essential for understanding the delivered dose in online adaptive radiotherapy (OART). The aim of this study is to quantify the dose accumulation accuracy and clinical significance of dose accumulation errors in pelvis treatments using the Ethos v1.1 and v2.0 systems.

**Method:**

Three pelvic CTs had anatomically realistic deformation vector fields (DVF) applied to create new modified images. These modified images were then used as treatment images in simulated OART treatment sessions, and the dose was accumulated by the Ethos system (*D(fx)_Ethos_*). The inverse of the applied (true) DVF was also used to accumulate the delivered dose (*D(fx)_True_*). The maximum applied DVF magnitude was compared to the maximum DVF magnitude calculated by the Ethos v1.1 and v2.0 systems. A 3D gamma analysis was performed between *D(fx)_Ethos_* and *D(fx)_True_*, and the clinical goals for each scenario were compared.

**Results:**

Large discrepancies in deformable registration magnitude were noted between the applied DVF and the Ethos DVF from both Ethos versions. All Ethos v1.1 results had 90% of points passing a 3%/2 mm gamma analysis, whilst Ethos v2.0 results did not reach this benchmark in all of the simulated scenarios. Results were particularly poor for the v2.0 prostate case. Inaccuracies in the dose accumulation process caused 6% of goals to change sufficiently such that the goal was reported as met or exceeded when it was not.

**Conclusion:**

The Ethos v1.1 dose accumulation system has been found to perform acceptably for the pelvic CTs tested in this study. For the Ethos v2.0 system substantial discrepancies in the dose accumulation were noted.

## 1 INTRODUCTION

In radiotherapy it is known that the planned dose does not exactly match the delivered dose due to a range of delivery‐based uncertainties.^1^, ^2^, ^3^ Nevertheless, due to the complexity of determining the delivered dose, it is rarely performed on a per fraction basis, and in most clinics, studies and clinical trials, the planned dose is assumed to be what was delivered. This simplification has sometimes been referred to as the static dose approximation.^4^

Online adaptive radiotherapy (OART) involves the creation of a new treatment plan for each treatment session that is optimized to the anatomy of the day. As the OART process involves the generation of a treatment plan on a different image set for each fraction, there is no single planning dose that can be assumed to be the delivered dose. In the case of OART, a dose accumulation method is required to assess the total delivered dose and is usually based on deformable image registration (DIR). DIR algorithms determine a deformation vector field (DVF) that links the anatomy in each voxel in one image to the voxel (or partial voxel) containing the same anatomy in the second image.^5^ DIR is required to account for non‐rigid changes in patient anatomy and can be used to also deform the dose grid for dose accumulation.

The Ethos platform (Varian Medical Systems, Palo Alto, CA) is an O‐ring gantry linear accelerator with software systems specifically designed to support OART, including an integrated dose accumulation system. For more information about the Ethos system and workflow see Archambault et al.^6^ For dose accumulation, the Ethos system uses an elastic deformation model, with a mutual information cost function with additional structure guidance constraints based on contoured structures, and B‐spline deformation regularization.^7^, ^8^ In Ethos, dose is deformed using direct dose mapping (DDM).^9^, ^10^ For a recent discussion on DDM compared with the energy/mass transfer (EMT)^11^, ^12^ method of deforming dose, refer to Murr et al.^13^

OART with Ethos thus far has been highly focused on the pelvic region^14^, ^15^, ^16^, ^17^, ^18^ as this is an area of clinical benefit, high image quality, and is well supported in current Ethos versions by pelvic artificial intelligence (AI) contouring models, allowing for more efficient treatments. Due to the relatively widespread use of Ethos in the pelvis, this study examines the accuracy of dose accumulation in male and female pelvic anatomy.

Accurate dose accumulation is important to several clinical workflows, allowing for better prospective prediction of toxicities, as well as greater understanding of dose‐response relationships when used in clinical trials. In the OART context it can also more accurately quantify the dosimetric changes relative to other treatment approaches, such that this resource‐intensive treatment technique^19^ can be targeted where greatest benefit exists. The accuracy of the Ethos dose accumulation algorithm has previously only been studied within a phantom,^20^ which may not be representative of behavior in patients. This study aims to investigate the accuracy of the Ethos OART dose accumulation system in both v1.1 and v2.0 platforms for anatomically realistic changes in pelvic patient CTs. The study also aims to quantify any errors in the dose accumulation process and determine their clinical significance.

## 2 METHOD

### 2.1 Dataset creation

Publicly available pelvic CT datasets that had already met ethics requirements for sharing were sourced to facilitate easier data sharing between the institutions collaborating on this study. Two prostate datasets from The Cancer Imaging Archive (TCIA).^21^ Prostate Anatomical Edge Cases repository^22^, ^23^ were selected for the study and used in accordance with the TCIA data usage policy. A gynecological dataset from the 2018 Elekta ProKnow (Elekta, Stockholm, Sweden) plan challenge was also used with permission from Elekta.

A range of anatomically realistic deformable registrations were applied to the CT images using the ImSimQA v4.3 software (OSL, Cardiff, UK) to create modified CT images (*CT_Mod_*). This software has previously been employed for this purpose in other studies^24^, ^25^ The deformations were chosen to represent common clinical scenarios, with a particular focus on bladder and rectum filling differences which are common in OART to the pelvis. Deformation magnitudes are described based on the following approximate guide: changes <5 mm are small, 5–10 mm as medium, and >10 mm as large. Where necessary, the field of view (FOV) of the images was adjusted such that the image center was located in the geometric center of the target region, this was to replicate cone beam CT (CBCT) images which are acquired with the image center aligned to the isocentre in the OART process. The deformable registration applied in each scenario is considered the ground truth and referred to as *DVF_True_*. The resolution of *DVF_True_* was 0.9–1.0 mm in the transverse plane and aligned with the CT slice spacing (2.0–2.5 mm) in the craniocaudal axis.

### 2.2 Simulated treatment delivery

The study was broken into two components, Part 1 tested the dose accumulation accuracy across a range of plan delivery parameters, and Part 2 tested the dose accumulation accuracy across a range of clinically representative anatomical changes.

The original undeformed CT images (*CT_Plan_*) were imported into both Ethos v1.1 (Part 1 and 2) and v2.0 (Part 2 only) treatment emulators, where the version numbers listed refer to the *Ethos Treatment Planning* application. The Ethos emulator is a test system that allows a simulated OART treatment to be carried out using the clinical Ethos software, but rather than acquiring a treatment CBCT on the linac, any CT or CBCT image can be imported as the treatment image. An online adaptive treatment plan was generated in both Ethos v1.1 and v2.0 using the same contours and planning directives between versions. Planning directives were matched as closely as possible, but some differences were needed due to version differences. The prostate plan was prescribed 60 Gy in 20 fractions (Fx) and the cervix plan was prescribed 45 Gy in 25 Fx. Planning objectives were based on eviQ guidelines^26^ with a 9‐field intensity modulated radiation therapy (IMRT) beam arrangement (except for the specified scenario described below in Part 1).

Each of the manipulated images were imported as treatment CBCTs in the emulator (both v1.1 and v2.0) and simulated treatments were performed. During each fraction, AI was used for structure contouring where available, then manual edits were made as required. During manual editing, structures were matched to the anatomy of the modified image, with anatomical boundaries (e.g. superior extent of rectum) matched to the structures used in the planning image. Treatments were performed in accordance with previously published workflows^27^ and were not time constrained. After generation of the plans, the *scheduled plan* was selected for treatment (except for the scenario described below in Part 1). In the Ethos workflow the *scheduled plan* is the plan generated during planning recalculated on the anatomy of the day, in contrast to the *adaptive plan*, which is reoptimized to the anatomy of the day. The *scheduled plan* was chosen to remove the additional variable of the re‐optimization process. Note that due to access limitations, experienced clinical staff from University of California San Diego performed the v2.0 treatments, whilst experienced clinical staff from Icon Group performed the v1.1 treatments.

#### 2.2.1 Part 1 – planning parameter impact

In Part 1, a single prostate patient was used with two simulated images: a modified anatomical scenario and a no change scenario. The modified anatomical scenario was simulated with a large expansion of the bladder. The “no change” scenario was simulated by applying no anatomical change to the CT, with all other steps unchanged, including loading and resampling in the ImSimQA software. For each of the two images in Part 1, four simulated fractions were performed in Ethos v1.1 generating four sets of dose distributions by different processes. The first simulated fraction (Part 1.1) was performed according to the workflow described above and used as a baseline for the following 3 simulated fractions. The second simulated fraction (Part 1.2) followed an identical process to fraction 1 and tested the consistency of the dose accumulation results. For the third simulated fraction (Part 1.3) the adaptive plan was selected, to test whether this had any effect on the dose accumulation accuracy. The fourth simulated fraction (Part 1.4) used a Volumetric Modulated Arc Therapy (VMAT) beam arrangement, to test the effect the beam arrangement had on dose accumulation accuracy.

#### 2.2.2 Part 2 – anatomical motion impact

In Part 2, eight different anatomical scenarios were included for the prostate case and 10 for the cervix case, as well as a “no change” scenario for each as described above. Each anatomical scenario was considered as a new fraction, with scenarios chosen to simulate a range of anatomical variations commonly seen over a patient's treatment course. The summated dose over all fractions was also analyzed, to simulate the total dose over a treatment course with day‐to‐day variations in anatomy.

### 2.3 Dose accumulation

After completion of the fraction, the Ethos system creates a deformable registration, *DVF_Ethos_*, between the treatment image (in this case *CT_Mod_*), and the planning CT (*CT_Plan_*). The transverse resolution of *DVF_Ethos_*, (1.8–3.9 mm in this study) is equal to 4 times the CT voxel size, with craniocaudal resolution 2–5 mm. The Ethos system then uses this registration to accumulate the scheduled plan dose from the fraction back to the planning CT, referred to as *D(fx)_Ethos_*. All data was then exported from the Ethos system.

**FIGURE 1:**
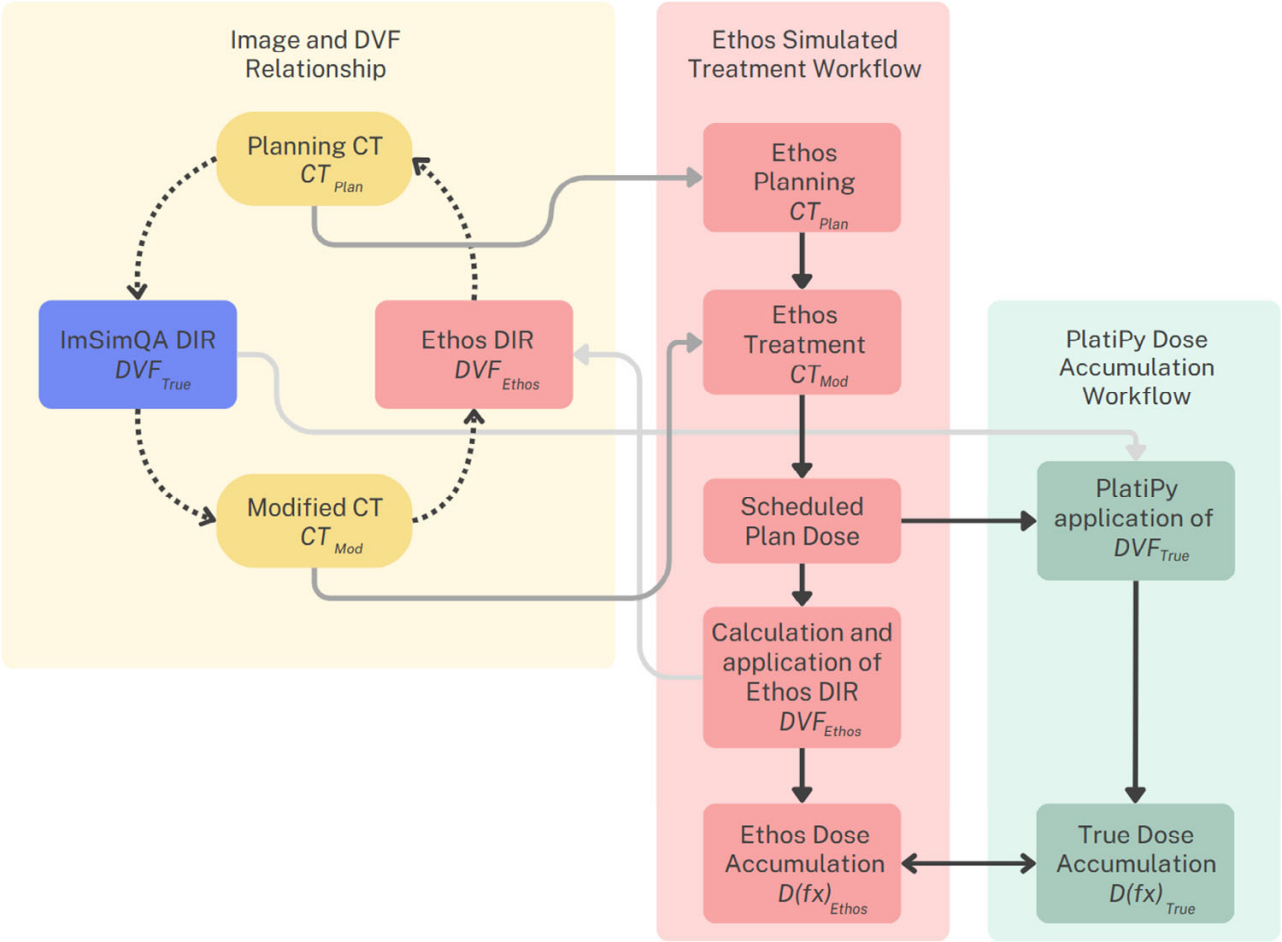
Schematic illustrating relationship of different steps and data objects in the study methodology. Dashed arrows indicate deformable registrations. Note that *DVF_True_* was used to create *CT_Mod_*, whilst *DVF_Ethos_* was created by the Ethos system to relate *CT_Mod_* to *CT_Plan_*.

A customized version of the open‐source software PlatiPy^28^ was used to deform the delivered fraction dose from *CT*_*Mod*_ to *CT*_*Plan*_ using the inverse of *DVF_True_*, referred to as *D(fx)_True_*. A schematic of the creation of *D(fx)_True_* and *D(fx)_Ethos_* is shown in Figure 1. In addition, for a given patient, all the fractions of *D(fx)_True_* and *D(fx)_Ethos_* were respectively summated to determine agreement over multiple fractions as described above.

### 2.4 Data analysis

PlatiPy was used to report the maximum deformation vector per structure from *DVF_True_* and *DVF_Ethos_*. The maximum was chosen as it indicates the range of motion seen. Dose accumulation accuracy was analyzed by performing a 3D gamma analysis^29^ between *D(fx)_True_* and *D(fx)_Ethos_* for each fraction and the summated fractions in the PTW VeriSoft software v7.2 (PTW, Freiburg, Germany). Results were reported using the AAPM TG218^30^ recommended patient specific QA criteria of 3%/2 mm, as well as 1%/1 mm and 5%/3 mm for additional information. Although not being used for patient specific QA in this study, the 3%/2 mm criteria was chosen as it represents a widely used threshold for clinically relevant errors. A 10% threshold dose was used for all gamma comparisons and percentage dose differences were calculated relative to the global maximum dose. To assess clinical significance of the changes seen in Part 2, clinical goals met for *D(fx)_True_* and *D(fx)_Ethos_* for each fraction and the summated fractions were analyzed. For these comparisons all doses were scaled to the prescribed fractionation and clinical goals based on the planning objectives were extracted from Eclipse v18.0 (Varian Medical Systems, Palo Alto, CA). As a quality check on contouring consistency between simulated treatments carried out on the same image in Ethos v1.1 and Ethos v2.0, Dice Similarity Coefficient (DSC) and Hausdorff Distance (HD) were also calculated using a script in Eclipse v18.0.

## 3 RESULTS

### 3.1 Deformable registrations—DVF magnitudes

The maximum deformation magnitudes per structure are shown for *DVF_True_*, *DVF_Ethos_* (v1.1) and *DVF_Ethos_* (v2.0) in Table 1. In the cases with deformations applied, the maximum true deformations ranged from 5.8 to 46.9 mm. In the “no change” scenarios small deformations were noted up to 1.1 mm, even though no structures were actively manipulated, possibly due to resampling effects from loading the images in the ImSimQA software and modifying the FOV. Note that the maximum vector reported does not necessarily occur in the same location between DVFs, and therefore a similar maximum vector does not necessarily indicate good agreement between DVFs.

**TABLE 1:** Maximum deformation magnitude per major structure, shown for *DVF_True_*, *DVF_Ethos_* (v1.1) and *DVF_Ethos_* (v2.0). For these data, voxels were assigned to structures based on the planning CT structure set. For *Part 1–Prostate* where multiple fractions were performed using the same image, results are shown for the baseline simulated fraction (Part 1.1). Where the discrepancy between *DVF_True_*, and *DVF_Ethos_* is greater than 10 mm, the result is highlighted in yellow.

			Body DVF maximum (mm)			PTV DVF maximum (mm)			Prostate/uterus DVF maximum (mm)			Rectum DVF maximum (mm)			Bladder DVF maximum (mm)		
Patient	Anatomical scenario	Fx/part	*DVF_True_*	*DVF_Ethos_* (v1.1)	*DVF_Ethos_* (v2.0)	*DVF_True_*	*DVF_Ethos_* (v1.1)	*DVF_Ethos_* (v2.0)	*DVF_True_*	*DVF_Ethos_* (v1.1)	*DVF_Ethos_* (v2.0)	*DVF_True_*	*DVF_Ethos_* (v1.1)	*DVF_Ethos_* (v2.0)	*DVF_True_*	*DVF_Ethos_* (v1.1)	*DVF_Ethos_* (v2.0)
Part 1 ‐ prostate	No change	1.1	1.1	35.8	–	0.3	20.8		0.2	10.1	–	0.0	35.8	–	1.1	8.0	–
	Bladder large expansion	1.1	46.9	35.4	–	28.6	24.3		7.6	12.9	–	25.5	35.4	–	46.9	24.9	–
Part 2 ‐ prostate	No change	1	0.0	7.6	19.9	0.0	3.8	19.7	0.0	3.3	19.2	0.0	7.4	17.1	0.0	4.6	19.9
	Bladder med shrink	2	9.9	13.0	30.5	3.3	5.5	17.5	2.2	3.3	16.8	2.0	7.5	22.3	9.6	13.0	30.5
	Bladder med expansion	3	11.2	12.8	27.0	3.5	5.0	15.9	1.3	4.9	15.9	7.6	7.8	27.0	11.2	12.7	18.3
	Bladder large expansion	4	36.5	26.9	44.6	10.9	6.3	19.8	3.7	4.1	19.7	32.0	17.9	39.2	35.5	26.2	28.1
	Rectum med shrink	5	5.8	9.2	20.4	4.3	3.7	18.3	3.8	3.7	17.7	5.8	9.2	20.4	1.3	4.0	19.5
	Rectum med expansion	6	7.5	7.2	28.7	6.4	6.8	19.3	5.6	6.3	18.7	7.5	7.2	24.4	2.8	4.3	28.7
	Rectum large expansion	7	11.7	9.3	23.5	8.6	8.5	16.2	7.7	8.5	16.2	11.7	9.1	18.7	7.7	6.6	23.5
	Bladder med shrink & Rectum med expansion	8	9.9	12.9	32.9	6.3	6.2	21.2	5.4	6.2	17.1	8.2	7.1	20.4	9.6	12.9	32.9
	Bladder med expansion & Rectum med expansion	9	11.1	12.9	22.3	6.4	6.8	17.8	5.6	6.1	17.8	10.2	7.2	22.2	11.1	12.7	19.4
Part 2 ‐ cervix	No change	1	1.0	38.9	25.6	1.0	21.4	6.1	1.0	12.2	5.7	0.1	15.9	25.6	0.2	16.0	4.9
	Bladder med shrink	2	9.0	39.0	20.7	8.5	18.1	18.6	1.7	11.1	8.9	0.8	14.7	20.7	9.0	9.3	18.6
	Bladder med expansion	3	10.3	39.1	26.5	9.7	21.7	12.3	4.1	12.7	12.3	1.2	16.4	26.5	10.3	8.7	9.7
	Bladder large expansion	4	15.5	39.3	31.3	14.5	24.1	21.0	7.8	12.8	11.9	3.7	8.1	23.0	15.5	18.7	24.4
	Rectum large shrink	5	10.4	39.3	31.0	10.4	22.7	26.1	8.3	7.6	12.7	10.3	18.6	31.0	1.6	5.7	9.3
	Rectum med shrink	6	6.8	39.1	26.7	6.8	20.5	17.2	5.3	6.7	10.9	6.8	17.3	26.7	1.3	4.9	9.2
	Rectum large expansion	7	13.8	39.1	23.8	13.7	21.5	16.7	9.6	11.9	12.4	13.8	12.5	23.8	2.8	12.5	13.1
	Bladder med expansion & Rectum med shrink	8	10.3	39.1	19.7	9.7	22.0	18.5	5.3	9.5	11.3	6.9	18.3	19.7	10.3	8.7	12.8
	Bladder med expansion & Rectum med expansion	9	10.3	39.0	23.9	9.8	20.0	10.0	4.9	5.9	8.8	7.2	13.7	23.9	10.3	8.9	9.5
	Uterus med expansion	10	8.1	39.2	26.6	8.1	17.7	19.8	8.1	11.3	17.8	6.7	14.0	26.6	4.6	7.0	8.7
	Uterus med shrink	11	7.3	39.1	37.4	7.3	22.7	37.4	6.9	13.3	37.4	4.6	13.8	26.3	3.0	3.0	5.4

### 3.2 Dose accumulation accuracy—gamma results

The dose accumulation accuracy gamma results for Part 1 are shown in Table 2. For Part 2 the results are shown in Table 3. In Part 1 all fractions had more than 90% of points passing the 3%/2 mm gamma analysis. In Part 2, apart from the v2.0 prostate patient, all fractions had more than 90% of points passing the 3%/2 mm gamma analysis.

**TABLE 2:** Gamma analysis results from Part 1 of the study, showing agreement between *D(fx)_Ethos_* and *D(fx)_True_* over a range of techniques/scenarios for a prostate case. Gamma results above 95% are highlighted in green, between 90% and 95% in yellow, and below 90% in red.

				Gamma results		
				Comparing tue dose with ethos v1.1 prediction [Dataset A: *D(fx)_True_* (v1.1), Dataset B: *D(fx)_Ethos_* (v1.1)]		
Part (Test)	Image modification scenario	Plan type	Adaptive or scheduled	1%/1 mm	3%/2 mm	5%/3 mm
1.1 (Baseline)	No change	IMRT	Scheduled	94.5%	97.8%	98.5%
1.2 (Consistency)				94.4%	97.7%	98.5%
1.3 (Adaptive plan)			Adaptive	93.9%	97.6%	98.8%
1.4 (VMAT)		VMAT	Scheduled	95.8%	98.5%	99.2%
1.1 (Baseline)	Bladder large expansion	IMRT	Scheduled	84.2%	91.5%	94.2%
1.2 (Consistency)				83.9%	91.4%	94.2%
1.3 (Adaptive plan)			Adaptive	84.2%	91.9%	94.6%
1.4 (VMAT)		VMAT	Scheduled	86.3%	93.3%	95.8%

**TABLE 3:** Gamma analysis results from part 2 of the study, showing agreement between *D(fx)_Ethos_* and *D(fx)_True_*, for a prostate and cervix patient across a range of clinical anatomical scenarios. Gamma results above 95% are highlighted in green, between 90% and 95% in yellow, and below 90% in red.

			Gamma results					
			Comparing true dose with ethos v1.1 prediction [Dataset A: *D(fx)_True_* (v1.1), Dataset B: *D(fx)_Ethos_* (v1.1)]			Comparing true dose with ethos v2.0 prediction [dataset A: *D(fx)_True_* (v2.0), dataset B: *D(fx)_Ethos_* (v2.0)]		
Patient	Image modification scenario	Fx	1%/1 mm	3%/2 mm	5%/3 mm	1%/1 mm	3%/2 mm	5%/3 mm
Part 2–prostate	No change	1	90.5%	98.6%	99.7%	81.7%	89.8%	93.6%
	Bladder med shrink	2	89.8%	97.6%	99.1%	79.8%	87.2%	91.0%
	Bladder med expansion	3	89.9%	98.9%	99.8%	81.8%	89.6%	93.2%
	Bladder large expansion	4	83.1%	96.8%	99.3%	77.8%	87.3%	91.0%
	Rectum med shrink	5	90.0%	98.7%	99.7%	81.9%	89.9%	93.7%
	Rectum med expansion	6	91.0%	98.5%	99.7%	79.8%	87.6%	91.4%
	Rectum large expansion	7	91.3%	98.7%	99.7%	83.7%	90.2%	93.5%
	Bladder med shrink & Rectum med expansion	8	90.9%	98.2%	99.3%	81.2%	87.8%	91.2%
	Bladder med expansion & Rectum med expansion	9	90.8%	99.3%	99.8%	82.2%	89.7%	93.4%
	Summation of fractions 1–9		91.9%	99.3%	99.8%	81.4%	88.8%	92.5%
Part 2–cervix	No change	1	93.9%	98.0%	99.0%	94.8%	99.1%	99.6%
	Bladder med shrink	2	93.7%	98.2%	99.1%	91.6%	98.5%	99.6%
	Bladder med expansion	3	92.7%	97.8%	98.9%	89.7%	98.1%	99.5%
	Bladder large expansion	4	89.1%	96.7%	98.6%	83.8%	94.8%	97.9%
	Rectum large shrink	5	93.2%	98.2%	99.2%	89.8%	96.5%	98.3%
	Rectum med shrink	6	93.9%	98.6%	99.3%	90.1%	96.9%	98.8%
	Rectum large expansion	7	88.8%	96.3%	98.4%	81.4%	92.4%	96.2%
	Bladder med expansion & Rectum med shrink	8	92.3%	98.3%	99.2%	88.7%	97.4%	99.2%
	Bladder med expansion & Rectum med expansion	9	92.4%	98.4%	99.3%	87.1%	97.0%	99.3%
	Uterus med expansion	10	91.7%	96.8%	98.3%	84.5%	92.5%	95.4%
	Uterus med shrink	11	94.1%	98.1%	99.2%	89.2%	96.9%	98.6%
	Summation of fractions 1–11		95.2%	98.7%	99.4%	91.3%	99.2%	99.8%

### 3.3 Clinical significance—clinical goals

The number of clinical goals met for *D(fx)_Ethos_* and *D(fx)_True_* for each fraction were analyzed. Goals that were met for *D(fx)_Ethos_*, but were not met for *D(fx)_True_*, or vice versa, were recorded in Table 4. This indicates that errors in the dose accumulation process on average cause 6% of goals to change sufficiently such that a goal is reported as met or exceeded when it was not. Of note, for the goals where discrepancies occurred:

‐ The Ethos accumulated dose was more likely to indicate that the goal was passing.
‐ None related to the CTV, 75% related to the PTV, and 25% related to organs at risk (OARs).

**TABLE 4:** Summary of clinical goals that did not agree between *D(fx)_Ethos_* and *D(fx)_True_* for each fraction.

Patient	Ethos version	Number of goals per fraction	Number of fractions analyzed	Total Number of goals that passed in *D(fx)_Ethos_* but failed on *D(fx)_True_*	Total Number of goals that failed in *D(fx)_Ethos_* but passed on *D(fx)_True_*	Percentage of goals that have different goal adherence between *D(fx)_Ethos_* and *D(fx)_True_*
Part 2–Prostate	v1.1	22	9	7	3	5%
Part 2–Prostate	v2.0	22	9	10	0	5%
Part 2–Cervix	v1.1	21	11	19	2	9%
Part 2–Cervix	v2.0	21	11	5	6	5%

Most pelvic OART treatments are delivered with multiple fractions, and therefore the summed dose will be more clinically relevant to outcomes and clinical trial data. The goals that were met for the summed dose, as well as the worst result for each goal across all fractions are shown in Tables 5 and 6.

**TABLE 5:** Clinical goals for the summation of all 9 prostate fractions, scaled to the total dose, displayed for *D(fx)_True_* and *D(fx)_Ethos_*, for Ethos versions 1.1 and 2.0. The worst value for each goal seen across all fractions is shown in brackets. Metrics where the summated dose did not meet the goal are highlighted in yellow, and those that did not meet the allowed variation in red.

			Ethos v1.1			Ethos v2.0		
Structure	Clinical Goal (and allowed variation)	Units	*D(fx)_True_*	*D(fx)_Ethos_*	Planned Dose	*D(fx)_True_*	*D(fx)_Ethos_*	Planned dose
CTV_6000	D0.03cc < 105% (109%)	Gy	61.9 (62.1)	61.7 (61.9)	62.2	61.9 (62.1)	61.7 (61.9)	62.1
CTV_6000	D95% > 100%	Gy	61.1 (61.0)	61.1 (61.0)	60.9	60.7 (60.7)	60.8 (60.8)	60.7
CTV_6000	V60Gy > 95% (93%)	%	99.6 (98.9)	99.9 (99.7)	99.4	100.0 (100.0)	100.0 (100.0)	100.0
PTV_6000	D0.03cc < 105% (109%)	Gy	62.2 (62.5)	62.2 (62.5)	62.4	62.3 (62.6)	62.4 (62.7)	62.9
PTV_6000	D95% > 100%	Gy	58.8 (54.8)	59.7 (57.9)	60.4	59.6 (54.4)	60.6 (60.1)	60.6
PTV_6000	V60Gy > 95% (93%)	%	89.6 (82.8)	93.6 (88.4)	97.0	93.3 (87.0)	98.0 (95.7)	98.8
Rectum	D0.03cc < 62.4 Gy (64.2 Gy)	Gy	59.3 (61.2)	60.4 (61.2)	60.4	60.9 (61.8)	61.3 (61.8)	61.0
Rectum	V60Gy < 3% (10%)	%	0.0 (0.7)	0.1 (1.0)	0.3	1.0 (2.5)	1.5 (2.8)	1.1
Rectum	V57Gy < 10% (15%)	%	1.4 (3.0)	2.1 (3.4)	3.3	3.3 (5.0)	2.8 (4.4)	3.4
Rectum	V52.8 Gy < 20% (30%)	%	4.6 (6.3)	5.0 (6.6)	6.9	5.9 (7.6)	4.1 (6.0)	5.8
Rectum	V50Gy < 22% (30%)	%	6.9 (8.5)	7.1 (8.7)	9.4	7.5 (9.2)	5.0 (7.0)	7.3
Rectum	V48.6 Gy < 30% (50%)	%	8.2 (9.7)	8.2 (9.8)	10.7	8.4 (10.0)	5.4 (7.6)	8.1
Rectum	V40.8 Gy < 35% (60%)	%	17.8 (19.7)	17.5 (19.4)	21.1	14.0 (15.5)	8.9 (11.6)	13.5
Rectum	V40Gy < 38% (45%)	%	19.2 (21.1)	18.9 (20.9)	22.5	14.8 (16.2)	9.6 (12.1)	14.3
Rectum	V30Gy < 57% (65%)	%	41.4 (42.8)	42.0 (44.5)	43.7	27.9 (28.9)	20.3 (25.0)	27.6
Rectum	V20Gy < 85% (90%)	%	59.6 (60.9)	61.8 (64.0)	59.7	45.6 (46.0)	40.4 (46.0)	45.7
Bladder	D0.03cc < 62.4 Gy	Gy	61.6 (61.9)	61.7 (61.9)	61.8	61.2 (61.6)	61.3 (61.5)	61.7
Bladder	V60Gy < 5% (10%)	%	2.1 (5.6)	2.8 (3.8)	3.2	1.6 (4.7)	3.8 (4.2)	4.3
Bladder	V48.6 Gy < 20% (25%)	%	8.5 (14.0)	7.4 (10.1)	8.9	7.9 (13.9)	6.9 (8.2)	10.7
Bladder	V40.8 Gy < 35% (50%)	%	13.0 (20.0)	10.9 (16.0)	13.2	11.9 (19.5)	8.5 (11.0)	14.7
Femur head & neck left	V40.8 Gy < 45% (50%)	%	0.0 (0.0)	0.0 (0.0)	0.0	0.0 (0.0)	0.0 (0.0)	0.0
Femur head & neck right	V40.8 Gy < 45% (50%)	%	0.0 (0.0)	0.0 (0.0)	0.0	0.0 (0.0)	0.0 (0.0)	0.0

**TABLE 6:** Clinical goals for the summation of all 11 cervix fractions, scaled to the total dose, displayed for *D(fx)_True_* and *D(fx)_Ethos_*, for Ethos versions 1.1 and 2.0. The worst value for each goal seen across all fractions is shown in brackets. Metrics where the summated dose did not meet the goal are highlighted in yellow.

			Ethos v1.1			Ethos v2.0		
Structure	Clinical goal	Units	*D(fx)_True_*	*D(fx)_Ethos_*	Planned dose	*D(fx)_True_*	*D(fx)_Ethos_*	Planned dose
CTVp	D0.03cc < 105%	Gy	46.1 (46.4)	46.1 (46.5)	46.4	45.9 (46.5)	45.9 (46.5)	46.1
CTVp	D95% > 100%	Gy	45.7 (45.5)	45.7 (45.5)	45.6	45.4 (44.8)	45.4 (44.8)	45.4
CTVp	D97% > 99%	Gy	45.7 (45.5)	45.7 (45.4)	45.5	45.3 (44.7)	45.4 (44.8)	45.3
CTVp	V45Gy > 95%	%	100.0 (100.0)	100.0 (100.0)	100.0	100.0 (82.3)	100.0 (88.7)	99.9
PTV	D0.03cc < 105%	Gy	46.2 (46.7)	46.2 (46.8)	46.7	46.3 (46.9)	46.2 (46.9)	46.8
PTV	D95% > 100%	Gy	44.2 (42.1)	44.5 (40.5)	45.0	44.3 (41.1)	44.6 (40.7)	45.1
PTV	D97% > 99%	Gy	43.7 (39.9)	44.1 (39.1)	44.8	43.7 (39.3)	44.1 (39.0)	44.8
PTV	V45Gy > 95%	%	88.6 (81.7)	89.3 (74.9)	95.5	88.9 (73.4)	91.4 (75.1)	96.0
Rectum	D0.03cc < 47.25 Gy	Gy	46.2 (46.5)	46.2 (46.6)	46.6	46.1 (46.7)	46.0 (46.7)	46.6
Rectum	V40Gy < 50%	%	46.1 (51.6)	48.0 (50.2)	47.2	42.6 (45.8)	41.1 (46.9)	43.4
Bladder	D0.03cc < 47.25 Gy	Gy	46.1 (46.6)	46.1 (46.7)	46.6	46.0 (46.4)	45.9 (46.4)	46.1
Bladder	V40Gy < 50%	%	41.6 (46.5)	40.3 (43.2)	40.7	41.2 (45.9)	39.0 (44.3)	41.2
Bowel Bag	D0.03cc < 47.25 Gy	Gy	46.2 (46.5)	46.1 (46.5)	46.6	46.2 (46.7)	46.1 (46.7)	46.8
Bowel	D0.03cc < 47.25 Gy	Gy	45.9 (46.2)	45.9 (46.3)	46.2	46.2 (46.6)	46.1 (46.5)	46.7
Bowel‐ CTVs	D0.03cc < 47.25 Gy	Gy	45.9 (46.2)	45.9 (46.3)	46.2	46.2 (46.6)	46.1 (46.5)	46.7
Bowel‐ CTVs	V45Gy < 50cc	cc	36.2 (43.4)	34.8 (52.7)	39.1	47.7 (56.0)	48.0 (70.4)	51.3
Bowel‐ CTVs	V40Gy < 100cc	cc	53.9 (59.5)	55.2 (67.6)	54.6	70.9 (77.6)	69.2 (89.0)	71.8
Femur Head & Neck Left	D0.03cc < 47.25 Gy	Gy	22.7 (23.5)	22.8 (23.5)	23.1	25.0 (26.2)	25.1 (26.3)	25.3
Femur Head & Neck Left	V30Gy < 15%	%	0.0 (0.0)	0.0 (0.0)	0.0	0.0 (0.0)	0.0 (0.0)	0.0
Femur Head & Neck Right	D0.03cc < 47.25 Gy	Gy	22.6 (23)	22.7 (22.9)	23.2	23.2 (24.1)	23.5 (24.3)	24.2
Femur Head & Neck Right	V30Gy < 15%	%	0.0 (0.0)	0.0 (0.0)	0.0	0.0 (0.0)	0.0 (0.0)	0.0

### 3.4 Contouring consistency—DSC and HD

As a quality control measure, DSC and HD were calculated between the contours generated during the simulated treatment in Ethos v1.1 and the contours generated on the same image during the simulated treatment in Ethos v2.0. The average and range of DSC and HD for each structure are shown in Table 7.

**TABLE 7:** Mean and range of DSC and HD comparing the contouring performed during simulated treatments in Ethos v1.1 with Ethos v2.0 for a given modified image set (*CT_Mod_*). Mean and range are calculated over 9 fractions for the prostate patient and 11 fractions for the cervix patient in part 2 of the study.

Patient	Structure	Mean DSC (Range)	Mean HD (Range) (mm)
Part 2 ‐ Prostate	CTV_6000/Prostate	0.900 (0.878–0.914)	4.4 (3.3–5.6)
	PTV_6000	0.921 (0.902–0.935)	4.6 (3.4–6.6)
	Bladder	0.958 (0.930– 0.968)	3.6 (3.2–5.3)
	Rectum	0.923 (0.800–0.940)	7.3 (4.7–16.5)
Part 2 ‐ Cervix	CTVp	0.898 (0.840–0.921)	5.7 (3.6–8.7)
	PTV	0.950 (0.930– 0.965)	5.5 (3.7–8.0)
	Bladder	0.857 (0.814–0.899)	4.7 (3.1–7.6)
	Bowel	0.919 (0.911–0.926)	6.3 (5.5–8.3)
	Rectum	0.888 (0.826–0.907)	8.9 (3.4–19.8)
	Uterus	0.884 (0.850– 0.921)	5.2 (3.8–6.8)

## 4 DISCUSSION

This study investigated the performance of two versions of a commercial dose accumulation algorithm using simulated anatomical changes in the pelvic region. This is an important step in the pathway to clinical implementation of dose accumulation. Findings indicate the potential for mischaracterization of anatomical changes which can result in changes in compliance with clinical goals, consistent with the phantom based results of Maraghechi et al.^20^

The maximum DVF data in Table 1 allows an understanding of the magnitude of the simulated anatomical changes and their effects on surrounding organs. Large discrepancies were seen between the maximum vector from *DVF_Ethos_* and *DVF_True_*. In particular, Ethos v2.0 frequently created DVFs with maximum magnitudes far larger than the applied deformation motion, this was noted for both the prostate and cervix case. In the Ethos v1.1 cervix patient, a large and consistent maximum vector of approximately 39 mm is seen for every fraction in the body structure, but not in any of the main organs at risk. This suggests that it may not have occurred in close proximity to the treatment area. By visualizing the DVF it was found that this was caused by inconsistency in the bowel contour between planning and treatment (cropping of the superior extent of the supplied bowel contour on the cervix v1.1 planning CT), which was not corrected by the staff member performing the simulated treatment because it was a large distance from the treatment area, but still resulted in a large vector in this area due to the DIR structure guidance. Similar effects were noted in other fractions, often due to inconsistency in the superior and inferior extent of a contour, for example, rectum. This highlights the importance of consistent contouring when using structure‐guided DIR approaches.

Gamma analysis was used to determine the accuracy of the dose accumulation. The Part 1 results shown in Table 2 were designed to test the methodological consistency and effect of different plan parameters on dose accumulation accuracy. First looking at the Part 1.1 no change scenario, it is interesting that even when very little simulated anatomical change was applied, *D(fx)_Ethos_* does not exactly match *D(fx)_True_*. This indicates that the motion seen in *DVF_Ethos_* in the no change scenario in Table 1, is affecting the dose distribution. The differences may be caused by variations in contouring between planning and treatment, the DIR algorithm itself, or even resampling effects due to the dose grid and DVF resolution.

Comparing results from Part 1.1 to Part 1.2, it is seen that results are quite consistent when repeated. The only differences expected in this consistency test is the effect of variations in manually edited auto‐contouring between subsequent fractions. Comparing Part 1.1 to Part 1.3, shows that the choice of adaptive or scheduled plan has very little effect on dose accumulation accuracy. Comparing Part 1.1 with Part 1.4, slightly higher gamma pass rates are observed with a VMAT beam arrangement. It is thought this is because near the patient surface if the defined beam edges seen in IMRT overlap with a DIR error, they can lead to gamma failures. In contrast for VMAT, due to the spread of beam entry angles there are no defined beam edges near the surface, so DIR errors in these regions generally do not lead to gamma failures. This is consistent with the work of Saleh‐Sayah et al.^31^ that suggested that geometric errors in DIR only become significant to dose accumulation in regions of steep dose gradients.

**FIGURE 2:**
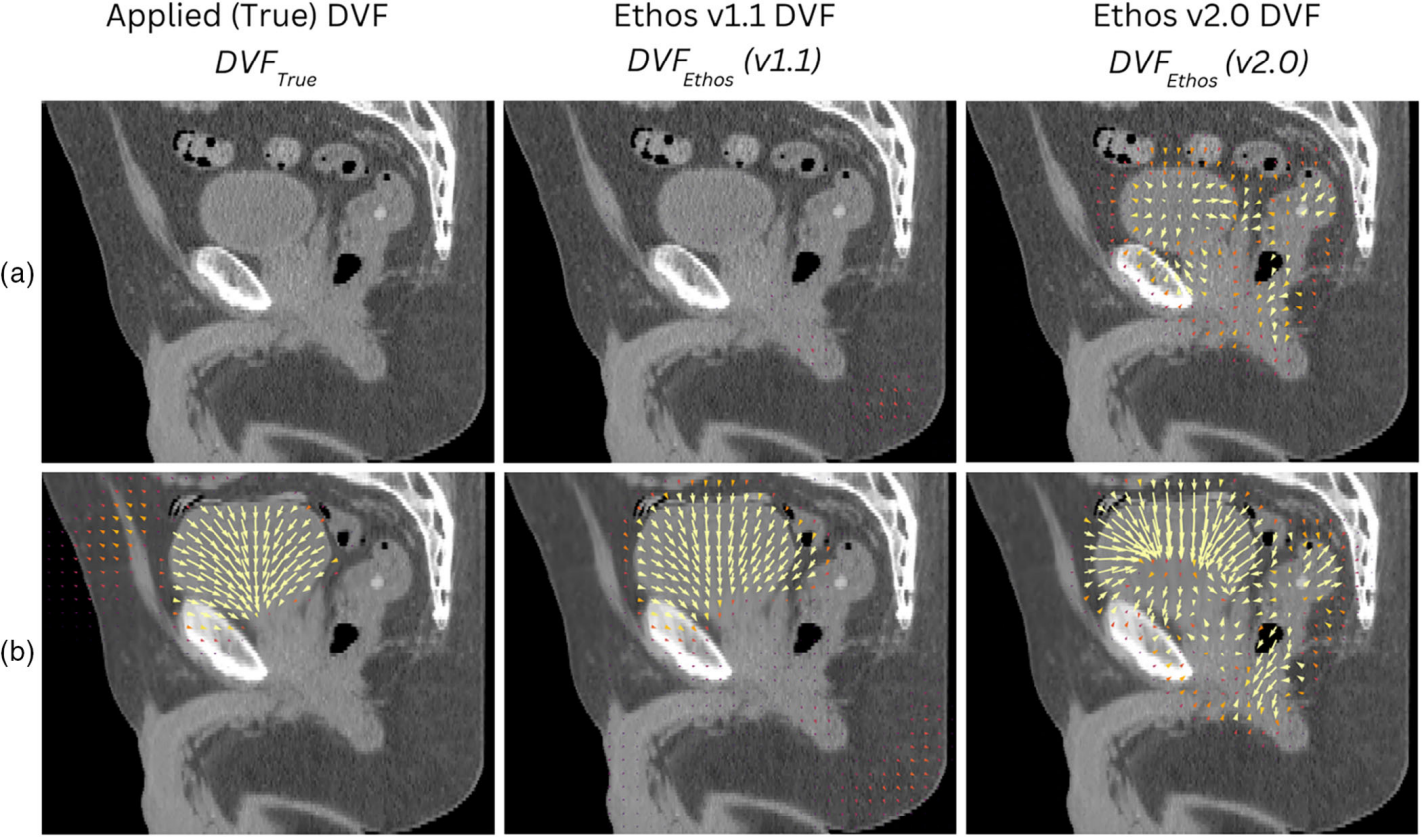
Sagittal visualization of the DVF overlaid on the modified CT. Row A displays DVFs from *Part 2 ‐ Prostate* Fx1, where no deformation was applied to the planning CT. Row B displays DVFs from *Part 2 ‐ Prostate* Fx4, where the bladder was expanded by a large amount. Note, *DVF_True_* is created in the opposite direction to *DVF_Ethos_*. For the purposes of this comparison, so that all vector directions are consistent between the images, the inverse of *DVF_True_* is displayed here. Also note all vector arrows are scaled to their actual size relative to the CT. Yellow arrows indicate the vector is greater than 10 mm, whilst a sliding color scale is used from 0 to 10 mm. Qualitatively, *DVF_Ethos_* (v1.1) appeared similar to *DVF_true_* while *DVF_Ethos_* (v2.0) was more inconsistent.

**FIGURE 3:**
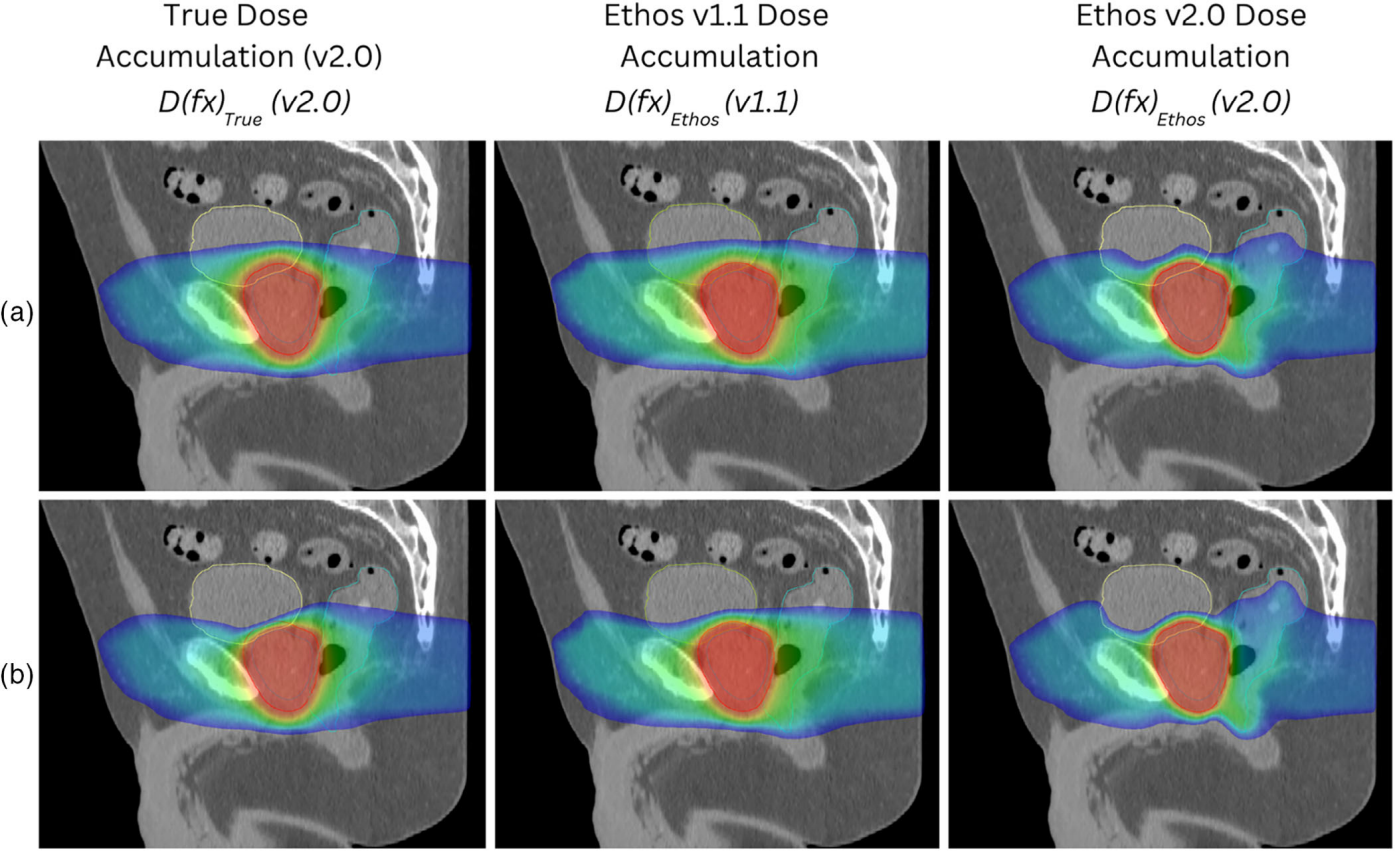
Sagittal visualization of the accumulated dose overlaid on the planning CT. Row A displays accumulated doses from *Part 2 ‐ Prostate* Fx1, where no deformation was applied to the planning CT. Row B displays accumulated doses from *Part 2 – Prostate* Fx4, where the bladder was expanded by a large amount. Note that *D(fx)_True_* differs between v1.1 and v2.0 because the planned dose distributions are different, although visually they look relatively similar. Only *D(fx)_True_* (2.0) is displayed in this figure.

Part 2 of the study aimed to simulate a range of realistic anatomical motions and their effect when summed over multiple treatment fractions. The dose accumulation accuracy results for Part 2 (shown in Table 3) found generally acceptable agreement between *D(fx)_Ethos_* (v1.1) and *D(fx)_True_* (more than 95% of points passed 3%/2 mm criteria gamma analysis). When *D(fx)_Ethos_* (v2.0) was compared to *D(fx)_True_*, similar results were observed for the cervix case, but substantially worse results were observed for the prostate case. Fractions with larger applied motions generally obtained lower gamma results than those with smaller motion or no change applied, although the differences were minor. For the v2.0 prostate case, all fractions failed to meet a 95% gamma passing criteria for all criterions tested. This suggests v1.1 is more accurate for dose accumulation, although further testing is needed. Reviewing *DVF_Ethos_* in Figure 2 shows that large differences occur in the vector field between versions. Of note, in the v2.0 “no change” scenario, vectors in opposing directions at the interface of the structures are seen. The DVF visualization suggests that a reduction has occurred in the relative strength of the regularization constraint used in the deformable registration algorithm in Ethos v2.0. The effects of the DVF on the dose are shown in Figure 3. The gamma analysis of the summed dose tends to be at the higher end of the results seen. This may be because random errors on a single fraction (e.g., due to inconsistencies in contouring) become less significant as more fractions are accumulated. These results suggest that determining the delivered dose can be a substantial source of error in the treatment delivery chain, and potentially a larger source of uncertainty than treatment delivery accuracy which would typically easily meet the 3%/2 mm gamma analysis criteria.^32^

It was found that errors in the dose accumulation process resulted in changes to which clinical goals are reported to be met in 6% of goals. Clinical goals are chosen to represent parameters of clinical relevance or interest and therefore are generally a good surrogate for clinically significant changes to the dose distribution. However, in this case most differences in goals were related to PTV coverage, which is just a tool to achieve CTV coverage, and may not be of particular concern in the OART and dose accumulation setting.

When the goals met for the summation of *D(fx)_True_* were compared to the summation of *D(fx)_Ethos_*, very few differences were noted except for the PTV coverage (seen in Tables 5 and 6). The one exception to this was the Prostate v2.0 case, where the true dose to the rectum and bladder was more than indicated by the Ethos dose accumulation. As seen with the dose accumulation accuracy results above, this indicates that as the number of fractions increase, the importance of dose accumulation errors on individual fractions decreases. Maximum dose goals for organs were stable between *D(fx)_True_* and *D(fx)_Ethos_*, though the correlation of their anatomical location is not evaluated. The worst‐case clinical goal across all fractions was largely consistent with the results for the summed dose: largest discrepancies were noted for the PTV and v2.0 rectum, bladder and bowel OARs.

The summed dose analysis in this study assumes that a range of different anatomical variations are seen during a treatment course. However, with hypofractionation, or patients that exhibit consistent shifts, for example due to lessening bladder control over the treatment course, or a simulation scan that is atypical for the patient, the worst‐case fraction results may be more indicative of the total dose over the treatment course.

With the exception of the *Part 2–prostate* v2.0 case, better agreement was seen between *D(fx)_Ethos_* and *D(fx)_True_*, than was seen between the planned dose and *D(fx)_True_*. This is likely because the errors in the DIR tend to be smaller than the errors in the static dose approximation, and therefore the implementation of dose accumulation is usually an improvement despite the presence of residual errors. This is supported by studies that have found that accumulated dose is a better predictor than planned dose for toxicities in prostate radiotherapy.^33^, ^34^

Table 7 gives information about the consistency of contouring between the simulated fractions in Ethos v1.1 and those performed in Ethos v2.0. Note that in the cervix case the initial bladder was empty, and therefore relatively small, leading to a lower expected DSC. The results indicate contouring was relatively consistent between v1.1 and v2.0, and that inconsistent contouring was not the main cause of the unusual vector fields noted previously in Ethos v2.0. The one exception to this is the larger HD distance found for the rectum, likely indicating that despite the best efforts of staff, inconsistency in the superior and inferior extent of rectum contouring occurred. This observation is consistent with what was noted from the results of Table 1.

Based on these results it appears consistency of contouring is very important for DIR accuracy, but not necessarily dose accumulation accuracy. Large discrepancies in contouring consistency were noted in the rectum in the Part 1 prostate, and the bowel in the Ethos v1.1 cervix case, resulting in large errors in the DVF, but these did not translate into dosimetric errors, likely because these were in areas of low dose gradient. Conversely good contouring consistency was noted visually in the Ethos v2.0 prostate case, but there was poor dose accumulation accuracy. For the v2.0 prostate case, the more irregular vector fields occurred in areas of dose gradient, it is suspected this led to the lower dose accumulation accuracy seen.

One limitation of this study is the images used to simulate CBCTs were actually deformed CT scans, and not true CBCTs. This meant they were likely higher quality than would typically be seen on standard Ethos linacs, except those equipped with a Hypersight imager.^35^ Another potential limitation of this method is that given it uses a DIR algorithm to create the “True” DVF, it is possible that the simulated deformations do not accurately represent real‐world changes in patient anatomy, and that DIR algorithms that are similar to the one used to create the modified images will better be able to replicate the applied DIR. A further limitation of this study is the limited patient cohort and range of anatomical scenarios. It is acknowledged that further testing is needed over a greater range of scenarios to confirm the results found here. In addition, all v1.1 treatments were performed by one institution, and all v2.0 treatments by another. Although every effort was made to ensure matching workflows were utilized, it is possible that systematic workflow differences occurred that could affect the results.

The results of this study highlight the importance of performing validation tests on dose accumulation software to understand its accuracy and limitations prior to clinical use. The development and integration of quality assurance methods within dose accumulation software, such as the ability to visualize the DVF, could facilitate these checks on a patient‐specific basis. Many authors have investigated the uncertainties that occur in deformable dose mapping and suggested quality assurance methods.^31^, ^36^, ^37^, ^38^, ^39^, ^40^, ^41^, ^42^, ^43^, ^44^

## 5 CONCLUSION

This study has tested the accuracy of the Ethos v1.1 and v2.0 software to accumulate dose in both female and male pelvic anatomy. It was found that there were marked differences in dose accumulation accuracy between Ethos v1.1 and v2.0, with substantially better results noted for Ethos v1.1 as determined by gamma analysis. This study found uncertainties in the dose accumulation process led to changes to whether a clinical goal was reported to be met for an average of 6% of the clinical goals. While further research is needed to confirm these results over a larger patient cohort, this study has demonstrated a methodology in which this can be achieved.

## AUTHOR CONTRIBUTIONS

Mikel Byrne, Xenia Ray, Kelly Kisling and Ben Archibald‐Heeren conceived of and designed the study, and delivered the simulated fractions. Mikel Byrne modified the images. Robert Finnegan and Mikel Byrne edited Platipy code and performed data analysis. All authors advised on data interpretation and presentation. Mikel Byrne drafted the study, and all authors reviewed the manuscript.

## CONFLICT OF INTEREST STATEMENT

Icon Group has a research agreement with Varian Medical Systems. Mikel Byrne, Xenia Ray, Kelly Kisling and Ben Archibald‐Heeren have received honoraria for presenting on behalf of Varian Medical Systems. Xenia Ray has a research agreement with Varian Medical Systems and is the recipient of consulting fees from KoRTUC. Robert Finnigan reports a relationship with SeeTreat Pty Ltd that includes consulting and advisory services.

## DATA SHARING

Modified data is unable to be shared due to licensing restrictions on the software used for image manipulation. The original unmodified images can be obtained from the public repositories: The Cancer Imaging Archive Prostate Anatomical Edge Cases repository [https://www.cancerimagingarchive.net/collection/prostate‐anatomical‐edge‐cases/] and Elekta ProKnow [https://www.elekta.com/products/oncology‐informatics/elekta‐one/real‐world‐outcomes/proknow/].
